# Off-axis biomechanical alterations and related interventions in medial knee osteoarthritis: a mini review

**DOI:** 10.3389/fresc.2026.1759442

**Published:** 2026-02-19

**Authors:** Zongpan Li, Raziyeh Baghi, Li-Qun Zhang

**Affiliations:** 1 Department of Physical Therapy & Rehabilitation Science, University of Maryland Baltimore, Baltimore, MD, United States; 2 Fischell Department of Bioengineering, University of Maryland College Park, College Park, MD, United States; 3 Department of Orthopaedics, University of Maryland Baltimore, Baltimore, MD, United States

**Keywords:** biomechanics, frontal, gait retraining, medial knee osteoarthritis, off-axis, rehabilitation, transverse

## Abstract

Medial compartment knee osteoarthritis (OA) is the most common form of knee OA and can be influenced by off-axis (frontal and transverse plane) biomechanics. Abnormalities such as varus malalignment, elevated knee adduction moment (KAM), dynamic varus thrust, altered step width, lateral trunk lean, reduced tibial rotation, and abnormal foot progression angle (FPA) contribute to excessive medial loading and are associated with symptom severity and structural progression. These modifiable factors present important targets for conservative management. This mini-review synthesizes current evidence on off-axis biomechanical impairments in medial knee OA and evaluates rehabilitation strategies designed to modify these mechanics. Gait retraining strategies, particularly personalized FPA modification, can reduce KAM and improve pain, with real-time biofeedback enhancing effectiveness. Valgus off-loader bracing alleviates pain related to medial knee OA and may be used as an adjunct for appropriately selected patients, especially when combined with practitioner guidance on brace fit and use. Lateral wedge insoles may provide small biomechanical benefits in a subset of individuals, while its effectiveness on symptom relief is not affirmative. Hip abductor strengthening reliably improves symptoms and function, although its load-modifying mechanisms and structural benefits remain unclear. Overall, current evidence supports a personalized, biomechanically informed approach targeting on off-axis biomechanics to managing medial knee OA. Future work should prioritize long-term randomized controlled trials and precision-based methods to identify individuals most likely to benefit from off-axis biomechanical interventions. Future clinical trials should also distinguish structural and functional off-axis biomechanical factors to optimize intervention effectiveness across distinct biomechanical phenotypes within medial knee OA.

## 1 Introduction

Medial compartment knee osteoarthritis (OA) is the commonest radiographic and symptomatic form of knee OA (1) and is associated with disproportionate loading of the medial tibiofemoral cartilage and subchondral bone (2). While sagittal-plane mechanics and muscle function are important (3–7), off-axis (frontal and transverse plane) biomechanics play a central role in determining medial load distribution during weight-bearing activities (8–11). The external knee adduction moment (KAM) is widely used as surrogate measure of medial compartment loading because they reflect the frontal-plane moment arm of the ground reaction force relative to the knee center (12, 13). Increased KAM and visible varus thrust during gait have been associated with symptoms and radiographic progression of medial knee OA (14–19), making off-axis mechanics attractive targets for conservative interventions. Off-axis biomechanical abnormalities in medial knee OA may arise from distinct underlying mechanisms. Distinguishing between structural and functional origins of off-axis mechanics is clinically meaningful, as interventions such as gait retraining, foot progression angle modification, bracing, and orthotic use are likely to exert differential effects depending on the underlying biomechanical phenotype. Accordingly, recognizing that medial knee OA encompasses multiple off-axis biomechanical phenotypes may support more individualized, mechanism-informed off-axis training and management strategies.

This review addresses two linked questions: (i) what off-axis biomechanical (structural and functional) abnormalities characterize medial knee OA and relate to disease progression, and (ii) which training or device-based interventions effectively modify off-axis mechanics and improve clinical outcomes? We summarize current evidence related to the two questions aiming to provide insights on effective management of medial knee OA for clinicians and researchers.

## 2 Methodology of literature selection

We conducted targeted searches in PubMed and Google Scholar using combinations of the terms: “frontal”, “transverse”, “axial”, “knee adduction moment”, “varus thrust”, “foot progression angle”, “gait retraining”, “valgus brace”, “lateral wedge”, “hip abductor strengthening”, “medial knee osteoarthritis”, and “biomechanics”. We prioritized systematic reviews, meta-analyses, randomized controlled trials (RCTs), and prospective cohort and biomechanical studies, particularly those that quantified frontal or transverse plane kinematics/kinetics or evaluated interventions aimed at modifying off-axis loading. Representative studies and syntheses are cited in the text. Where possible, we emphasize recent evidence on personalized/precision approaches to gait retraining and device selection.

## 3 Off-axis biomechanical abnormalities in medial knee OA

### 3.1 Frontal plane biomechanics

Static varus alignment increases the medial moment arm and shifts load toward the medial compartment during stance. Varus malalignment leads to elevated medial tibiofemoral joint loads, which are linked to disease progression in medial compartment knee OA (20–23). It also predicts the onset of medial knee OA (23, 24). Moreover, increased medial inclination of the proximal tibia, reflecting coronal-plane tibial displacement, has been associated with elevated external KAM in individuals with advanced medial knee OA (25). Collectively, these findings indicate that patients with medial knee OA exhibit static structural alterations that are closely linked to disease severity and progression, and which may represent important targets for therapeutic intervention. Dynamic varus thrust—a sudden increase in varus angulation during weight acceptance—further amplifies medial loading transiently. Varus thrust observed during walking has been associated with a greater varus angle and increased peak varus angular velocity in stance (26), and it relates to clinical symptoms and structural damage characteristic of medial knee OA (27–31). Individuals with medial knee OA also exhibit increased medial joint laxity and reduced joint stiffness, potentially contributing to joint instability and symptoms (32–34). Additionally, wider step width (35–37) and lateral trunk lean (38–42) have been associated with reductions in frontal-plane knee loading, such as the KAM or medial knee joint contact force. The external KAM is widely used as a surrogate for medial compartment loading (12, 13). Individuals with medial knee OA commonly demonstrate elevated KAM, which has been linked to the development and progression of symptoms and structural deterioration (14–19). Moreover, hip abductor weakness was commonly observed in patients with knee OA (43), which is believed to affect frontal knee kinetics and may contribute to disease progression (44, 45). Taken together, the evidence indicates that patients with medial knee OA display a spectrum of frontal-plane functional biomechanical alterations—including dynamic varus thrust, step width, lateral trunk lean, elevated external KAM, and hip abductor weakness—that represent potential targets for effective management of the disease.

### 3.2 Transverse plane biomechanics

Both imaging and gait studies indicate that tibial rotational mechanics are altered in individuals with medial knee OA, although such structural and functional changes vary with disease severities (46–51). Imaging evidence suggests that the direction of tibial rotation abnormalities differs across stages of medial knee OA: increased tibial internal rotation has been observed under weight-bearing in early OA (50), whereas posterior tibial translation with relative external rotation—implying restricted knee extension—has been reported in advanced medial OA (49). In addition, preliminary work by Potti et al. proposed that a subset of medial knee OA may be characterized by loss of transverse-plane internal rotation, with relative proximal tibial external rotation occurring concurrently with reduced sagittal-plane extension and lateral tibial displacement in the frontal plane (51). Gait analyses demonstrate reduced axial tibial rotation excursion in knee OA, with medial knee OA specifically characterized by diminished tibial internal rotation or a bias toward external rotation during walking (46–48). Together, these findings suggest that medial knee OA is associated with altered static tibial rotation and constrained transverse-plane motion during gait, although the direction and magnitude of these restrictions appear to differ across disease stages. The reduction in internal tibial rotation has been hypothesized to represent a compensatory adaptation aimed at mitigating mechanical stimuli to surrounding tissues that might otherwise provoke knee pain (52, 53). A recent study introduced the pain threshold angle as a quantitative measure of mechanically induced knee pain during tibial internal rotation and found that smaller pain threshold angles were associated with more severe clinical symptoms; additionally, individuals with symptomatic medial knee OA demonstrated impaired proprioception in tibial rotation (54). Foot progression angle (FPA) (toe-out/in foot) is corresponded to tibial rotations and related to knee loads (10, 11). Hence, modifying FPA has become a commonly used gait-retraining strategy for managing knee OA (55).

## 4 Interventions that modify off-axis mechanics

### 4.1 Gait retraining: modifications of trunk lean, knee thrust, step width, and FPA

Gait retraining, including the modifications on ipsilateral trunk lean, medial knee thrust, step width, and FPA, has been used for the treatment of medial knee OA by reducing external KAM (56–58). Individual studies reported that ipsilateral trunk lean (39, 40, 59), medial knee thrust (60), greater step width (8, 35) can reduce knee loading and may produce acute improvements in knee symptoms. However, high-quality RCTs are needed to rule out potential placebo effects and to evaluate long-term benefits for symptom relief and structural protection. Moreover, these gait modifications (e.g., lateral trunk lean) may increase energy expenditure and produce uncertain biomechanical consequences at other joints (e.g., ankle, hip, trunk), which should be considered in clinical prescription (61).

Adjusting FPA alters the mediolateral position of the center of pressure and the frontal-plane lever arm of the ground reaction force, thereby reducing KAM (10, 11). A recent systematic review and meta-analysis concluded that, among individuals with medial knee OA, toe-out gait reduces the second peak external KAM and KAM impulse (55). A more recent study introduced a robotic-controlled stepping trainer capable of determining individualized FPA targets to optimize external KAMs (9). In addition, Uhlrich et al. (2022) employed a personalized approach for prescribing FPA modifications, increasing the proportion of individuals who may benefit from such an intervention (62). In a subsequent RCT, the authors demonstrated that personalized FPA modification delivered with real-time biofeedback resulted in acute improvements in knee pain, reductions in knee loading, and the potential to slow OA progression (63). Taken together, these findings suggest that personalized gait retraining delivered with real-time biofeedback or coaching can produce durable changes in gait mechanics and yield short-term improvements in pain, although long-term adherence and structural outcomes remain to be established.

### 4.2 Valgus off-loader bracing

Valgus bracing applies a corrective moment that shifts joint loads laterally, thereby offloading medial cartilage; some designs may also provide proprioceptive or neuromuscular benefits that contribute to symptom relief (64). Results from a systematic review concluded that evidence support the use of valgus off-loader bracing as an effective treatment for alleviating pain for medial knee OA, while its effect on function and stiffness remains inconclusive (65). More recent work suggests that integrating sensor technology into standard bracing for knee OA has the potential to further improve clinical outcomes (66). Despite the effectiveness of valgus off-loader bracing for symptom relief, several limitations warrant consideration. Studies report variable effect sizes, user discomfort, inconsistent long-term adherence, and insufficient long-term RCT evidence to determine whether bracing meaningfully alters structural disease progression. Importantly, although complex knee-bracing interventions are generally viewed as acceptable by first-line practitioners (e.g., physiotherapists), initial training in personalized brace selection, along with ongoing support and mentorship, is essential for enhancing practitioners' self-efficacy in delivering these interventions (67).

### 4.3 Lateral wedge insoles

Lateral wedge insoles modify hindfoot inversion/eversion and shift the center of pressure laterally, thereby reducing the frontal-plane moment arm and lowering the external KAM. Evidence regarding the effectiveness of lateral wedge insoles for treating medial knee OA has been evaluated in several systematic reviews and meta-analyses (68–73). Lateral wedge insoles can have small effect on knee biomechanics (e.g., reductions in knee adduction angle, KAM, KAM impulse), while it appears ineffective at attenuating structural changes in patients with medial knee OA (68, 69, 73). Although an overall pooled results showing the use of lateral wedge insoles is related to reduced knee pain, no such significant association was found when specifically using a neutral insole as the comparator (70–72). The divergence between immediate biomechanical effects and variable clinical outcomes suggests that conventional lateral wedge insoles may benefit only a subset of patients rather than serving as a universally effective intervention. Additionally, potential discomfort and downstream effects on ankle and hip mechanics pose concerns for widespread clinical implementation. More recently, Sabet et al. (2025) introduced a novel footwear design—combo slipper socks with an integrated lateral wedge insole. Using a within-subject experimental design, the authors reported improved comfort and greater knee pain reduction compared with conventional sandals incorporating lateral wedge insoles in female patients with bilateral medial compartment knee OA (74). Future RCT with extended follow-up are needed to determine whether advanced lateral wedge designs can enhance symptom relief and provide long-term structural protection while maintaining comfort in individuals with medial knee OA.

### 4.4 Hip abductor strengthening

Weakness of the hip abductors is believed to be related to increased dynamic knee adduction loading by failing to adequately control pelvic drop (44, 45). Recent systematic reviews of RCTs have concluded that hip abductor strengthening improves symptoms and functional outcomes (75, 76). Another RCT by Almeida et al. (2022) reported that hip abductor and hip adductor strengthening produce comparable improvements in pain, function, and quality of life (77). Evidence from prospective cohort study reported that hip abductor weakness was associated with worsened knee pain in females with strong knee extensors, suggesting knee extensor strengthening is important, but might not be sufficient, to prevent pain worsening (78). A recent RCT further demonstrated that, relative to quadriceps strengthening, hip abductor strengthening yielded superior effects on pain reduction and daily functioning (79). Thus, hip abductor strengthening reliably improves symptoms and function and should be incorporated into exercise-based management. However, the hypothesized mechanism whereby hip abductor strengthening reduces external knee loads has not been supported by current evidence (80). Additionally, the effectiveness of hip abductor strengthening for structural protection in knee OA remains unconfirmed.

## 5 Practical clinical recommendations

Current evidence indicates that off-axis biomechanical factors—including varus alignment, elevated external KAM, varus thrust, altered FPA, reduced tibial rotation, and hip abductor weakness—represent viable targets for clinical management of medial knee OA. Several conservative interventions can be considered in practice. Personalized gait retraining, particularly FPA modification delivered with real-time biofeedback, has shown consistent short-term improvements in pain and reductions in medial knee loading, and may be especially beneficial when individualized to a patient's biomechanical profile. Adjustments in trunk lean, step width, or medial knee thrust may acutely reduce KAM, although these strategies require careful monitoring due to potential increases in energy expenditure or unintended effects on adjacent joints. Valgus off-loader bracing alleviates pain related to medial knee OA and may be used as an adjunct for appropriately selected patients, especially when combined with practitioner guidance on brace fit and use. Lateral wedge insoles may provide small biomechanical benefits in a subset of individuals, while its effectiveness on symptom relief is not affirmative. Hip abductor strengthening reliably improves symptoms and function and should be incorporated into exercise-based management, although its biomechanical mechanisms remain uncertain.

## 6 Gaps in knowledge and future research directions

Despite promising advances, notable gaps remain in understanding and optimizing off-axis biomechanical interventions for medial knee OA. High-quality, long-term RCTs are needed to determine whether gait-retraining strategies, valgus bracing, and lateral wedge insoles produce sustained symptom relief, promote long-term adherence, or meaningfully alter structural disease progression. The biomechanical mechanisms linking hip abductor strengthening to changes in knee loading remain inconclusive, and evidence regarding its capacity to slow structural deterioration is lacking. For gait modifications such as trunk lean, step width, or knee thrust, the potential adverse effects on energy cost and multi-joint biomechanics require further investigation. Additionally, the heterogeneity of clinical responses to lateral wedge insoles and bracing underscores the need for improved prescreening tools to identify responders. Emerging technologies—such as robotic-controlled devices, wearable-integrated braces, and advanced footwear designs—show promise for personalizing interventions, but require validation in larger, long-term clinical trials. Future research should prioritize precision-medicine approaches that integrate patient-specific biomechanics, symptom profiles, and sensor-based feedback to optimize treatment selection and maximize therapeutic benefit. In addition, the use of artificial intelligence–based markerless motion analysis to guide personalized gait retraining holds promise for potential broad implementation in community settings and may offer a cost-effective approach to knee OA management (81, 82). Moreover, most included studies quantify off-axis mechanics during gait or weight-bearing tasks without explicitly differentiating structural malalignment from functional or dysfunctional movement. This distinction is clinically relevant because off-axis interventions such as gait retraining, FPA modification, bracing, and insoles may differentially benefit individuals depending on whether abnormal mechanics are structural/functional or originate from varus knee dysfunction. Hence, it is important to identify phenotype-based classification distinguishing off-axis structural changes from dysfunctional, direction-specific motion loss for medial knee OA. High-quality longitudinal and RCTs evaluating whether early identification and treatment of structural or dysfunctional off-axis mechanics can modify symptoms or disease trajectory of medial knee OA.

## 7 Conclusion

Off-axis biomechanical factors contribute meaningfully to the progression and symptoms of medial knee osteoarthritis and present viable targets for conservative intervention. Evidence supports short-term benefits of personalized gait retraining, valgus bracing, lateral wedge insoles, and hip abductor strengthening for reducing pain and knee joint loading in selected individuals. However, long-term adherence, structural outcomes, and consistent treatment response remain uncertain. Future work should emphasize individualized, biomechanically informed, phenotype-targeted approaches supported by rigorous long-term trials to optimize treatment effectiveness.
